# Breastfeeding in panhypopituitarism: a case report, review of the literature, and management checklist

**DOI:** 10.1186/s13006-026-00859-0

**Published:** 2026-06-16

**Authors:** Yael Derdikman Ofir, Anne Eglash, Kerstin Uvnäs Moberg, Esther Tahover, Irit Hochberg

**Affiliations:** 1 Section of Medical Oncology, Yale School of Medicine, 333 Cedar St. New Haven, New Haven, CT 06510 USA; 2 Department of Family and Community Medicine, University of Wisconsin School of Medicine and Public Health, Madison, WI USA; 3 Department of Animal Health and Welfare, Swedish University Agriculture, Skara, Sweden; 4 Department of Oncology, Assuta Medical Center, Ramat HaHayal, Tel Aviv Israel; 5 Faculty of Life Sciences, Bar Ilan University, Ramat Gan, Israel; 6 Endocrinology Unit, Hillel Yaffe Medical Center, Hadera, Israel; 7 Bruce Rappaport Faculty of Medicine, Technion – Israel Institute of Technology, Haifa, Israel; 8 Institute of Endocrinology and Metabolism, Rambam Health Care Campus, Haifa, Israel

**Keywords:** Panhypopituitarism, Hypopituitarism, Breastfeeding, Lactation, Intranasal oxytocin, Prolactin

## Abstract

**Background:**

Panhypopituitarism is a rare endocrine disorder characterized by deficiency of multiple pituitary hormones. Because prolactin and oxytocin are essential for lactation, breastfeeding is often presumed to be impossible in affected women, and guidance on lactation in this population is scarce.

**Methods:**

We conducted a literature search to identify reports describing breastfeeding outcomes in women with hypopituitarism or panhypopituitarism and examined clinical, obstetric, and treatment-related factors (including prior pituitary surgery and radiation therapy) potentially associated with breastfeeding success.

**Case study:**

We report the case of a 34-year-old pregnant woman with panhypopituitarism in Israel who established and maintained a sustained partial breastfeeding relationship following delivery. Lactation support included intranasal oxytocin administered before each breastfeed, use of a homemade nursing supplementer, galactagogues, and regular pumping. Breastfeeding was maintained for 6.5 months with infant formula supplementation.

**Results of literature review:**

We identified reports of lactation outcomes in 69 parous women with pituitary hormone deficiencies, 20 of whom were reported to have breastfed. In an exploratory multivariable logistic regression analysis incorporating demographic, medical, and labor-related variables, prior radiation therapy appeared to be associated with a lower likelihood of breastfeeding.

**Conclusions:**

This case and literature review suggest that breastfeeding may be possible in some women with panhypopituitarism, given individualized preparation and multidisciplinary support. The use of intranasal oxytocin in this context is hypothesis-generating and warrants further study. We propose a management checklist to assist clinicians in supporting individuals with panhypopituitarism who wish to breastfeed.

**Clinical trial registration:**

Not applicable.

**Supplementary Information:**

The online version contains supplementary material available at 10.1186/s13006-026-00859-0.

## Background

Hypopituitarism is an uncommon endocrine disorder (incidence of 4.2 cases per 100,000 per year and prevalence of 45.5 cases per 100,000) characterized by partial or complete deficiency of pituitary hormones due to a variety of etiologies, including congenital factors, trauma, brain hemorrhage, tumors, systemic hemorrhage during labor and infection [1]. Panhypopituitarism, a more severe form of the condition in which all pituitary hormones are affected, is even less common.

Women with hypopituitarism frequently experience difficulties achieving pregnancy due to deficiencies in gonadotropins and other pituitary hormones. Conception often requires assisted reproductive technologies, ranging from ovulation induction with gonadotropins to in vitro fertilization, along with close endocrinologic follow-up [2]. Owing to the low prevalence of hypopituitarism, reports on peripartum outcomes in women with this condition are limited [3–15]. Furthermore, the topic of lactation in women with panhypopituitarism is virtually absent from the scientific literature, with only brief mentions of a small number of patients, mostly stating that lactation was not initiated and that prolactin levels were low [3, 4, 7, 12–43]. This is not surprising given that the anterior pituitary hormone prolactin and the posterior pituitary hormone oxytocin, which are essential for breast milk production [44] and letdown [45, 46]^,^ respectively, are often deficient in these women, hindering the onset of lactation.

We present a case of a 34-year-old parturient woman with panhypopituitarism after treatment for craniopharyngioma who successfully achieved partial breastfeeding. We conducted a comprehensive literature search for reports on breastfeeding status in parturient women with partial or complete hypopituitarism and used a multivariable logistic regression model to examine the effects of different variables on breastfeeding. Recognizing the need for practical support, we conclude with a suggestion for a structured management checklist to guide clinicians in facilitating breastfeeding for pregnant women with panhypopituitarism to optimize care and outcomes.

## Case presentation

A 34-year-old White gravida 1 para 0 woman with a history of panhypopituitarism presented at 31 weeks’ gestation to a specialized outpatient lactation clinic in northern Israel, providing lactation consultant-led counseling, with the aim of preparing for breastfeeding after delivery, following discussion with her endocrinologist. Her medical journey began at the age of 28 when she was diagnosed with an adamantinomatous craniopharyngioma suprasellar stalk tumor. She underwent a craniotomy via the pterional approach (a lateral skull base surgical approach through the temporal region), during which a small piece of the tumor was intentionally left on the pituitary stalk [47]. This decision was due to the low malignant potential of the tumor and the patient’s desire to maintain hormonal function. Despite this, she developed panhypopituitarism immediately after the operation, including arginine vasopressin deficiency (AVP-D; formerly termed central diabetes insipidus – DI [48]), central hypoadrenalism, central hypothyroidism, hypogonadotropic hypogonadism and growth hormone (GH) deficiency, as well as hypothalamic obesity. She was treated with desmopressin, hydrocortisone, levothyroxine, GH, and oral contraceptives. The residual tumor on the pituitary stalk was further treated with a course of fractionated stereotactic radiotherapy. Her prepregnancy prolactin level was above normal (59.4 ng/mL, normal range 2.77–29.1 ng/mL). The mildly increased prolactin level was not treated since she was asymptomatic.

At the age of 33, she conceived after ovulation induction with human menopausal gonadotropin (HMG) and intrauterine insemination (IUI). Her pregnancy was complicated by an eruption of shingles on the left V1 dermatome (the ophthalmic branch of the trigeminal nerve, involving the periocular region) at week 8 and mild gestational hypertension.

**Fig. 1:**
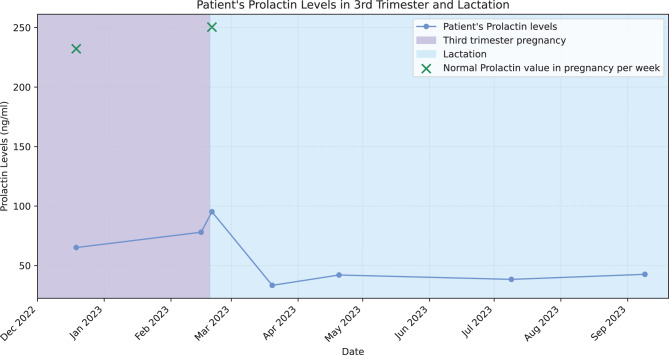
Patient serum prolactin levels in the third trimester of pregnancy and lactation. legend: patient serum prolactin levels measured during the third trimester of pregnancy and throughout the lactation period. Reference prolactin levels in healthy women at week 28 of pregnancy and on the first day postpartum are shown as green X symbols [49–51]. Blood samples obtained postpartum and during breastfeeding were collected after suckling. Shaded areas indicate the third trimester of pregnancy and the lactation period

Despite a clear understanding that hormonal deficiencies may not enable full breastfeeding, she expressed a strong desire to breastfeed and sought guidance from lactation physicians and consultants during pregnancy. Her breasts enlarged during the 2^nd^ and 3^rd^ trimesters. At 34 weeks’ gestation, antenatal colostrum expression was demonstrated by a lactation consultant during a prenatal counseling visit, primarily to teach manual expression techniques and assess feasibility. A small amount of colostrum (one drop) was expressed. During a follow-up visit at 36 weeks’ gestation, colostrum expression was again demonstrated, with one to two drops expressed. These brief demonstrations were intended as preparation for early postpartum lactation rather than as a routine antenatal expression regimen. Her prolactin levels, as noted above, were higher than normal before pregnancy, did not increase and were lower than expected during pregnancy and lactation [49–51], as shown in Fig. 1.

Oxytocin is necessary for milk ejection during lactation, and there are no available clinical tests for oxytocin because of its very short biological half-life [20]. Thus, it was assumed based on her AVP-D that she had some degree of oxytocin deficiency [12, 52].

As her delivery date approached, an elective cesarean section (c-section) was planned upon the patient’s request. This decision was made since a high incidence of peripartum complications and c-sections (emergent and elective) was reported in pregnant women with panhypopituitarism. After spontaneous rupture of membranes and the appearance of contractions, she underwent a semi-elective cesarean birth at 37 0/7 weeks gestation, without complications. She gave birth to a healthy male weighing 3284 g. Immediately after delivery, the newborn was placed skin-to-skin on her chest while on the operation table. The patient managed to initiate breastfeeding within the first hour postpartum in the post anesthesia care unit with the help of a board-certified lactation consultant nurse.

The patient breastfed with the aid of a nursing supplementer starting with the first latch. This homemade device is composed of a 5 french-wide 40 cm long tube connected on one end to a syringe or bottle with infant formula and taped on the other end to the breast, lined up with the nipple [53]. The initial use of the nursing supplementer required assistance from another person for syringe refilling, but later, she learned to use the formula bottle and handle the supplementer, allowing her to breastfeed independently (Additional file 1).

On the second day postpartum, pharmacotherapy with an oxytocin solution (Grindex 10 IU/mL) was started via the intranasal route via the MAD Nasal™ Intranasal Mucosal Atomization Device [54], with a dosage of 2 IU per use [45, 55–60]. These modes and methods of oxytocin administration were chosen since the intranasal route is preferred for milk ejection during lactation and is accessible at home and because commercial intranasal oxytocin spray was discontinued over two decades ago. Initially, the patient noted that the use of intranasal oxytocin before every session of breastfeeding facilitated milk ejection, particularly in the early days and weeks postpartum. However, after one month, there was no observable difference that could be attributed to oxytocin administration, and when tested, breastmilk continued to flow by hand expression without it. At that point, she decided to discontinue oxytocin.

The milk volume was not sufficient for exclusive breastfeeding, and her baby received supplementary infant formula. There is no clinically available recombinant human prolactin [61–63], and as she had elevated prolactin before pregnancy, indicating the presence of functionally remnant lactotrophs, she tried several galactagogues [58] known to increase prolactin levels and milk production in healthy women, which together noticeably increased milk production. The galactogogues included domperidone [64], which was initiated a month postpartum at 20 mg three times a day and reduced to 10 mg three times daily due to headaches. Herbal galactagogues, including goats’ rue, torbangun, and fenugreek, were also used. Both the goats’ rue and torbangun were discontinued after one month because of a slight increase in liver transaminases, which normalized after cessation [58].

As GH reportedly binds the prolactin receptor [65, 66] and acts as a galactagogue [67–71]^,^ she reinitiated her prepregnancy GH treatment of 0.4–0.6 mg a day.

In addition to breastfeeding with a homemade nursing supplementer and galactagogue administration, the patient pumped milk every 3 hours to stimulate milk production. Regular pumping did help with a slight increase in the expressed milk volume, from 2 to 3 mL to 4–7 mL per pumping session. Although prenatal lactation counseling had been provided, structured postpartum lactation follow-up was not continuous during the first several weeks after delivery. Until 5.5 weeks postpartum, the patient experienced severe bilateral nipple pain during breastfeeding. After formal lactation follow-up was re-established, persistent nipple pain was evaluated and infant ankyloglossia was diagnosed. A scissor frenotomy was performed by a lactation physician in an outpatient setting, followed by standard post-procedure stretching exercises. Following frenotomy, nipple pain improved substantially, allowing the patient to enjoy breastfeeding and increasing her motivation to continue.

Throughout her breastfeeding journey, the patient received close monitoring and support from a multidisciplinary team of an endocrinologist, a breastfeeding medicine physician, her primary care physician, and lactation consultants. After three months of galactagogue use, occasional pumping, and breastfeeding with aid from the nursing supplementer, she discontinued using the supplementer and continued breastfeeding for 10–15 minutes on each breast, followed by bottle supplementation with infant formula.

In this patient’s case, it is important to address the safety of prescription drugs during lactation. Each of the patient’s regular medications, including desmopressin [72, 73], hydrocortisone [74, 75], GH [76, 77] and levothyroxine [78–80]^,^ has been deemed safe for breastfeeding according to the LactMed and e-lactancia databases. The patient’s low milk production minimized the amount of medication to which the infant was exposed. Despite menopausal-like hot flashes beginning at 4 months postpartum, she elected to postpone resuming estrogen replacement until breastfeeding cessation. Overall, the patient breastfed her baby for 6.5 months and discontinued breastfeeding after the infant began biting during breastfeeding.

From her point of view, the patient found the opportunity to provide herself and her son with the unique benefits of breastfeeding, being in close contact with her son and feeding him with as much of her own milk as possible, to be an experience that she discovered somewhat serendipitously. She was determined to explore the possibilities, and despite the challenges, the experience was gratifying for her, and she summarized that the nurturing bond with her son made every effort worthwhile. This patient-reported experience illustrates the potential personal significance of breastfeeding beyond nutritional considerations, highlighting the relational and emotional dimensions that may motivate individuals with complex medical conditions to pursue breastfeeding.

## Methods

### Methodology of literature search and data collection

Our search strategy involves a combination of ‘hypopituitarism’ or ‘panhypopituitarism’ with one of the following terms: ‘lactation’, ‘nursing’, or ‘breastfeeding’ in PubMed and Google Scholar. The literature search was conducted between July 2022 and November 2024 and yielded only two directly relevant papers [42, 43]. We found brief mentions, typically a single line, of breastfeeding outcomes in papers discussing high-risk pregnancies in women with these conditions. To extract valuable insights, we identified articles describing pregnancy and childbirth in women with hypopituitarism or panhypopituitarism and systematically documented all cases, including mentions of infant feeding methods (breastfeeding, bottle feeding, or any combination thereof). We recorded the characteristics of the patients, pregnancies, births, newborns, and methods of infant feeding for each patient. There was no mention of the utilization of lactation aids, neither pharmacologically nor physically, such as the nursing supplementer. For patients with Sheehan syndrome, only pregnancies occurring after the pituitary insult and formal diagnosis were included. Breastfeeding outcomes from the index delivery that led to the diagnosis were excluded.

We sent emails to all corresponding authors of the papers inquiring whether they had additional information on prolactin levels before, during or after pregnancy; whether women who did not breastfeed attempted breastfeeding at all; and whether pharmacological interventions or lactation devices were used to aid breastfeeding. Regrettably, only one author responded that she had no additional information, and the rest did not reply.

## Statistical analysis

### Data preprocessing and statistical analysis

Missing value processing: Continuous variables were filled in with mean values. In our dataset, the mean birth weight was 3014 grams. There was one missing value for the following variables: surgery, radiation, conception method, cortisol, thyroid, and GH deficiencies; 13 missing values for birth complications; and 9 missing values for birth weight, which were filled in by the mean value [81] (see Additional file 2).

To eliminate duplicates resulting from either multiple deliveries for the same patient or from twin pregnancies, data for women with more than one delivery were taken from the first of the reported deliveries, as we noticed that women who did not breastfeed following their first delivery did not breastfeed after their subsequent deliveries. Additionally, data for women who had a twin pregnancy were reported only once in our dataset.

We used IBM SPSS Statistics (version 27) to analyze the data. A multivariable logistic regression model was fitted, and overall model significance was assessed using a likelihood ratio test (reported in SPSS as the Omnibus test of model coefficients) [82].

## Results

Our literature search yielded 36 reports on 69 women with multiple pituitary hormone deficiencies or panhypopituitarism who delivered a total of 87 live newborns (including our patient) [3, 4, 7, 12–43]. Their main characteristics are shown in Table 1, while full information can be found in Additional file 2.

**Table 1:** Characteristics of women with hypopituitarism or panhypopituitarism who achieved live births, as reported in the literature

Variable	n	Percentage
**Cause of panhypopituitarism**		
Sheehan’s syndrome	18	26
Craniopharyngioma	17	25
Chromophobe adenoma	5	7
Genetic	7	9
Prolactinoma	4	6
Agenesis/aplasia	4	6
Cushing’s disease	3	4
Macroadenoma	3	4
Sella tuberculoma	1	1
Pituitary stalk interruption syndrome	1	1
Iatrogenic	1	1
Histiocytosis X	1	1
Granular cell tumor	1	1
Acromegaly	1	1
Lymphocytic hypophysitis	1	1
Abscess	1	1
**Hormone deficiency**		
AVP deficiency	26	38
Cortisol deficiency	48	70
Thyroid deficiency	56	81
GH deficiency	15	22
**Mode of Conception**		
Spontaneous conception	20	29
In vitro fertilization	10	15
Ovulation induction	38	55
NA	1	2
**Delivery and labor complications**		
Vaginal delivery	30	44
Cesarean section	39	57
Emergency cesarean section	13	19
Induced vaginal delivery	9	13
Delivery complications	15	22
**Other**		
Twin pregnancy	7	10
Successful breastfeeding	20	28
Radiation therapy	13	19

Abbreviations: AVP, arginine vasopressin; GH, growth hormone; NA, not available

Table 1. Main characteristics of 69 women with hypopituitarism or panhypopituitarism who achieved live births, as reported in the literature, including the current case. The table summarizes the underlying etiologies, hormonal deficiencies, conception methods, delivery modes and complications, and selected clinical outcomes. Percentages are rounded to whole numbers and calculated based on available data for each variable.

Overall model significance was assessed using a likelihood ratio test (reported in SPSS as the Omnibus test of model coefficients). The multivariable logistic regression model included age at pregnancy, etiology of hypopituitarism, treatment history (surgery and radiation therapy), hormonal deficiencies, mode of conception, mode of delivery, birth weight, and delivery complications.

The overall model was not statistically significant [χ^2^(16)=23.13, *p* = 0.110], as expected given the small sample size.

The classification model predicted successful breastfeeding in 91.5% of cases and unsuccessful breastfeeding in 55% of cases. Radiation therapy was the only variable that reached nominal statistical significance in association with a lower likelihood of breastfeeding (*p* = 0.046). Table 2 presents all variables included in the analysis.

**Table 2:** Variables included in the multivariable logistic regression analysis of breastfeeding outcomes

Variable	Significance
**Age at pregnancy**	0.624
**Cause of hypopituitarism**	
Tumor vs no tumor	0.670
Acquired vs genetic	0.658
Sheehan vs other causes of hypopituitarism	0.617
Craniopharyngioma vs other causes of hypopituitarism	0.177
Surgery for pituitary condition	0.601
Radiation therapy for pituitary condition	0.046
**Hormone deficiency**	
AVP/ADH deficiency	0.090
Cortisol deficiency	0.117
Thyroid deficiency	0.460
GH deficiency	0.900
**Mode of Conception** (Spontaneous vs other)	0.260
**Mode of Delivery**	0.851
**Birth weight**	0.109
**Delivery complications**	0.182

Abbreviations: AVP, arginine vasopressin; ADH, antidiuretic hormone; GH, growth hormone

Table 2. Multivariable logistic regression analysis evaluating factors associated with successful breastfeeding among women with hypopituitarism or panhypopituitarism (*n* = 69).

## Discussion

There is an assumption that successful breastfeeding is impossible for women with panhypopituitarism due to combined posterior and anterior pituitary hormone deficiencies, and pregnant women with panhypopituitarism are usually discouraged from attempting breastfeeding or preparing for lactation. The case reported presents a unique instance that challenges this assumption, suggesting that partial breastfeeding may be achievable in some women with panhypopituitarism with individualized preparation and support.

Our patient, despite her panhypopituitarism, demonstrated a strong commitment to breastfeeding and sought extensive support from lactation specialists throughout her pregnancy and postpartum periods. Her success in breastfeeding was facilitated by a combination of preparation and planning that started during pregnancy and included manual expression, preparation for the innovative use of intranasal oxytocin postpartum, and support from a multidisciplinary team of lactation professionals. The patients’ efforts postpartum included immediate skin-to-skin contact after cesarean section delivery, colostrum expression, the use of oxytocin and galactagogues, the use of a nursing supplementer, and regular pumping. These efforts enabled the patient to establish and maintain a successful breastfeeding relationship with her baby, albeit with formula supplementation.

The use of intranasal oxytocin, involving repurposing a formulation intended for intravenous administration for intranasal use, was undertaken to approximate the effects of the previously available intranasal oxytocin spray (Syntocinon), which has since been discontinued. Intranasal oxytocin may have contributed to breastfeeding in this case, although its specific contribution cannot be determined from a single uncontrolled case, and further investigation is needed.

Close monitoring and support from a multidisciplinary team of lactation professionals played a crucial role in her breastfeeding journey. This case highlights the importance of individualized assessment and consultation with healthcare professionals when considering medication use during breastfeeding, especially in complex cases such as this.

Our comprehensive literature search revealed 69 women with pituitary hormone deficiency for whom information on breastfeeding was reported, 20 of whom breastfed. Notably, there is no information on whether women who did not breastfeed attempted breastfeeding, and it is possible that some were discouraged from initiating breastfeeding by their physicians, who anticipated that breastfeeding would be unsuccessful.

A multivariable logistic regression analysis was performed to assess the association between 12 clinical and demographic variables and breastfeeding outcomes, and previous radiation therapy was the only variable associated with unsuccessful breastfeeding. The relatively small number of cases, owing to the low prevalence of the condition and even lower reporting rate in the medical literature, limited the statistical power to identify or exclude the effect of additional factors. Accordingly, these findings should be considered exploratory and intended to stimulate further reports and studies in larger cohorts.

The extremely sparse information on prolactin levels in the collected cases (summarized in Additional file 2) is insufficient to determine whether circulating prolactin levels reliably predict breastfeeding success. In the present case, prolactin levels remained lower than expected during pregnancy and lactation, yet partial breastfeeding was achieved, highlighting the complexity of hormonal and non-hormonal factors involved.

This study has several limitations. First, due to the absence of standardized indexing for breastfeeding outcomes in women with hypopituitarism or panhypopituitarism, direct database searches identifying all relevant lactation cases were not feasible. Consequently, breastfeeding-related information was extracted from broader reports describing pregnancy outcomes in women with pituitary hormone deficiencies, and some published cases may have been missed. Second, the number of reported cases was small, limiting statistical power and generalizability. Third, information regarding prolactin levels during pregnancy and lactation was sparse and inconsistently reported across studies, limiting assessment of the relationship between prolactin levels and breastfeeding outcomes. Finally, the literature search was conducted through November 2024, and therefore more recently published cases were not included. The regression analysis should therefore be considered exploratory and hypothesis-generating.

## Conclusions

Further research and additional reported cases are needed to understand the specific factors or treatments that may enable successful breastfeeding in women with panhypopituitarism and provide insights for the management of breastfeeding in these individuals. Specifically, the potential for benefitting from the use of oxytocin and recombinant prolactin [63] is worth exploring. We hope that our case report, literature review, and proposed management checklist may serve as a practical resource for clinicians and support individuals with panhypopituitarism who wish to attempt breastfeeding.

### Suggested checklist for breastfeeding preparation and management for individuals with panhypopituitarism

The following checklist offers a structured approach for healthcare professionals to support individuals with panhypopituitarism in their breastfeeding journey. Collaborative care and proactive planning are crucial.

#### Medical consultation

1. People who wish to attempt breastfeeding should engage a lactation physician and lactation consultant during pregnancy to provide specialized guidance on breastfeeding challenges specific to panhypopituitarism. Lactation experts and endocrinologists should collaborate to understand the individual’s specific condition and its implications for breastfeeding.

#### Hormonal assessments

1. Consider measuring and reporting serum prolactin levels during pregnancy, preferably in the mid-third trimester, to assess whether levels are appropriately rising. As prolactin concentrations normally increase throughout gestation, measurement at this time may help identify low levels while still allowing time to plan postpartum lactation support strategies.
2. An additional prolactin measurement within the first postpartum week may provide further information regarding lactation potential.
3. Reference prolactin levels in healthy women at comparable late-pregnancy and early postpartum time points are shown in Fig. 1.
4. At present, the predictive value of prolactin levels for breastfeeding success in people with panhypopituitarism remains unclear, and additional data are needed.

#### Antenatal preparation

1. Realistic expectations should be set, and the potential benefits of feeding at the breast, independent of milk volume, should be emphasized, even when supplementation is required [83].
2. The use of breast expression techniques to stimulate colostrum expression may reassure people in whom milk is present.
3. Provide training on hand expression and the benefits of using a breast pump. Advice to have a breast pump available.
4. The importance of immediate skin-to-skin contact to foster bonding and encourage the initiation of breastfeeding in the first hour postpartum should be emphasized.
5. People should be guided to establish a regular pumping routine to maintain and potentially increase milk production.
6. The concept and potential benefits of using a nursing supplementer over a bottle should be introduced, all required parts should be prepared in advance, and early use should be recommended if supplementation is needed.

#### Pharmacological support

1. The potential benefits and administration methods of intranasal oxytocin should be discussed, ensuring that the individual understands that this use is off label. If they still wish to try it, provide a prescription prepartum to enable use shortly after labor.
2. The prescription of galactagogues to increase milk production may be considered early in the postpartum period, including during the first postpartum week, particularly in individuals with known risk factors for low milk production. The timing should be individualized based on clinical assessment and the individual’s preferences. Potential side effects and contraindications should be carefully considered, and if galactagogues are used, complete blood counts and basic metabolic panels should be monitored. In the present case, galactagogues were initiated later in the postpartum course due to non-clinical circumstances.
3. Complete a baseline electrocardiogram prior to initiating domperidone, particularly in individuals with cardiac risk factors or concomitant QT-prolonging medications, to assess the corrected QT (QTc) interval and exclude significant baseline prolongation.

#### Social support System

1. Encourage the individual to establish a close support system, including their partner, family, and friends, emphasizing the importance of emotional and practical support.

## Electronic supplementary material

Below is the link to the electronic supplementary material.

Supplementary material 1

Supplementary material 2

## Data Availability

All the data analyzed during this study are included in this published article and its additional files (Additional file 2).

## Abbreviations

ADH: Antidiuretic hormone
AVP-D: Arginine vasopressin deficiency
C-section: Cesarean section
DI: Diabetes insipidus
g: gram
GH: Growth hormone
HMG: Human menopausal gonadotropin
IU: International unit
IUI: Intrauterine insemination
mg: milligrams
mL: milliliter
QTc: Corrected QT interval

## Declarations

### Permission to reproduce material from other sources

All figures, tables, and materials in this article are original. No permissions were required for reproduction.
