# Supplementary material for: Breastfeeding in panhypopituitarism: a case report, review of the literature, and management checklist
Source: Int Breastfeed J. 2026 Jun 16;21:73. doi: 10.1186/s13006-026-00859-0 (PMC13445676; doi:10.1186/s13006-026-00859-0)
Supplement: Supplementary file 1 [file 13006_2026_859_MOESM2_ESM.docx]

| Count | AUTHOR | CASE | MOM_serial_number | BABY_serial_number | TWIN | Age_at_pregnancy | Diagnosis | Surgery | Radiation | G | P | A | conception | AVP-D | Cort Deficiency | Thyroid Deficiency | GH Deficiency | pregnancy regime | PRL_NON_PREGNANT | PRL_THIRD_TRIMESTER | PRL_AT_DELIVERY | Mode_of_delivery | EMERGENCY_CS | INDUCED_DELIVERY | Birth_weight | SEX | WEEK_GESTATION | COMPLICATION_DELIVERY | SUCCESFUL_BREASTFEEDING | Pharmacological means | Lactation devices |
| --- | --- | --- | --- | --- | --- | --- | --- | --- | --- | --- | --- | --- | --- | --- | --- | --- | --- | --- | --- | --- | --- | --- | --- | --- | --- | --- | --- | --- | --- | --- | --- |
| 1 | Index case | 1 | 1 | 1 | F | 34 | CRANIOPHARYNGIOMA | T | T | 1 | 1 | 0 | OI | T | T | T | F | De, H, Thy | 59.4 | 78 | 95.22 | CS | F |  | 3284 | M | 37 | F | T | T | T |
| 2 | Overton 2002 | 1 | 2 | 2 | F | 32 | PROLACTINOMA | F | T | 1 | 1 | 0 | OI | T | T | T | F | Br, Thy, H, De | NA | NA | 5.38 | CS | T |  | 2040 | F | 38 | NA | F | NA | NA |
| 2 | Overton 2002 | 2 | 3 | 3 | F | 29 | CRANIOPHARYNGIOMA | T | F | 3 | 1 | 2 | OI | F | T | T | F | Psl, Thy | NA | NA | 31.58 | CS | T |  | 2540 | M | 34 | T | F | NA | NA |
| 2 | Overton 2002 | 2 | 3 | 4 | F | 29 | CRANIOPHARYNGIOMA | T | F | 4 | 2 | 2 | OI | F | T | T | F | Psl, Thy | NA | NA | NA | CS | T |  | 2650 | F | 35 | T | F | NA | NA |
| 2 | Overton 2002 | 3 | 4 | 5 | F | 34 | CRANIOPHARYNGIOMA | T | F | 1 | 1 | 0 | OI | T | T | T | F | CA, Thy, De | NA | NA | 20.21 | CS | T |  | 3450 | M | 38 | F | T | NA | NA |
| 2 | Overton 2002 | 3 | 4 | 6 | F | 34 | CRANIOPHARYNGIOMA | T | F | 2 | 2 | 0 | OI | T | T | T | F | CA, Thy, De | NA | NA | NA | CS | F |  | 2620 | F | 38 | NA | T | NA | NA |
| 2 | Overton 2002 | 4 | 5 | 7 | F | 38 | SELLA TUBERCULOMA | F | F | 1 | 1 | 0 | OI | T | T | T | F | Thy, Psl, De | NA | NA | NA | CS | F |  | 3245 | M | 38 | NA | T | NA | NA |
| 2 | Overton 2002 | 6 | 6 | 8 | F | 31 | CUSHING | T | F | 2 | 1 | 1 | OI | F | T | T | F | H, Thy | NA | NA | <3.76 | CS | F |  | 2560 | M | 38 | NA | F | NA | NA |
| 2 | Overton 2002 | 8 | 7 | 9 | F | 33 | CRANIOPHARYNGIOMA | T | F | 1 | 1 | 0 | OI | T | T | T | F | H, Thy, De | NA | NA | <2.35 | CS | T | F | 2580 | M | 39 | T | F | NA | NA |
| 2 | Overton 2002 | 8 | 7 | 10 | F | 33 | CRANIOPHARYNGIOMA | T | F | 2 | 2 | 0 | OI | T | T | T | F | H, Thy, De | NA | NA | NA | CS | F |  | 2960 | M | 39 | NA | F | NA | NA |
| 2 | Overton 2002 | 9 | 8 | 11 | F | 42 | CHROMOPHOBE ADENOMA | T | F | 1 | 1 | 0 | OI | F | T | T | F | Psl, Thy | NA | NA | 2.49 | CS | T |  | 3980 | M | 40 | NA | F | NA | NA |
| 3 | Franks S 1977 | 1 | 9 | 12 | F | 36 | PROLACTINOMA | T | F | 1 | 1 | 0 | S | F | F | F | F | None |  | 15 | 15 | V |  | F | NORMAL | NA | TE | F | T | None | None |
| 4 | Volz 2002 | 1 | 10 | 13 | F | 34 | CRANIOPHARYNGIOMA | T | T | 1 | 1 | 0 | OI | T | T | T | F | Thy, H, De |  | SUB-NORMAL | SUB-NORMAL | V |  | F | 3450 | F | 37 | F | F | NA | NA |
| 5 | Van Zundert SKM 2021 | 1 | 11 | 14 | F | 30 | APLASIA | F | F | 1 | 1 | 0 | OI | T | T | T | F | Thy, H, De |  | NA | NA | CS | T | T | 3515 | M | 38 | F | F | NA | NA |
| 6 | Zhang J 2022 | 1 | 12 | 15 | F | 28 | PSIS | F | F | 1 | 1 | 0 | OI | F | T | T | T | Thy, H, GH | NA | NA | 3.25 | CS | F |  | 2860 | M | 36.4 | F | F | NA | NA |
| 7 | Shinar 2015 | 1 | 13 | 16 | F | 28 | CRANIOPHARYNGIOMA | T | T |  |  |  | IVF | T | T | T | F | GL, Thy, De | NA | NA | 0.28 | V |  | F | NA | NA | 39 | T | F | NA | NA |
| 7 | Shinar 2015 | 2 | 14 | 17 | F | 31 | MACROADENOMA | T | T |  |  |  | IVF | T | T | T | F | GL, Thy, De | NA | NA | 0.15 | CS | T | F | NA | NA | TE | T | F | NA | NA |
| 7 | Shinar 2015 | 3 | 15 | 18 | F | 26 | IARTOGENIC | F | T |  |  |  | NA | T | NA | NA | NA | NA | NA | NA | Undetectable | CS | T | F | NA | NA | NA | T | F | NA | NA |
| 7 | Shinar 2015 | 4 | 16 | 19 | F | 24 | HISTIOCYTOSIS X | F | T |  |  |  | IVF | T | T | T | F | GL, Thy, De | NA | NA | 0.12 | V |  | F | NA | NA | NA | T | F | NA | NA |
| 8 | Aulinas 2021 | 1 | 17 | 20 | F | 35 | CUSHING | T | F |  |  |  | OI | T | T | T | T | GH, Gn, TSH, ACTH, AVP-D | NA | NA | NA | V |  | T | 2495 | F | 37.4 | F | F | NA | NA |
| 8 | Aulinas 2021 | 2 | 18 | 21 | F | 36 | GRANULAR CELL TUMOR | T | F |  |  |  | OI | T | T | T | T | GH, Gn, TSH, ACTH, AVP-D | NA | NA | NA | V |  | T | 3330 | M | 42.1 | F | T | NA | NA |
| 8 | Aulinas 2021 | 3 | 19 | 22 | F | 34 | CRANIOPHARYNGIOMA | T | F |  |  |  | S | F | F | T | T | GH, Gn, TSH | NA | NA | NA | CS | F |  | 3170 | F | 36.3 | F | T | NA | NA |
| 8 | Aulinas 2021 | 5 | 20 | 23 | F | 36 | PROLACTINOMA | T | F |  |  |  | OI | F | F | T | T | GH, TSH, PCOS | NA | NA | NA | CS | T |  | 4040 | F | 42.2 | F | F | NA | NA |
| 8 | Aulinas 2021 | 6 | 21 | 24 | F | 42 | PROLACTINOMA | T | F |  |  |  | OI | F | F | T | T | GH, TSH, PCOS | NA | NA | NA | CS | F |  | 4100 | F | 40.4 | F | F | NA | NA |
| 8 | Aulinas 2021 | 7 | 22 | 25 | F | 29 | AGENESIS | F | F |  |  |  | OI | F | F | F | T | GH, Gn | NA | NA | NA | V |  | F | 3195 | M | 40 | F | F | NA | NA |
| 8 | Aulinas 2021 | 9 | 23 | 26 | F | 34 | AGENESIS | F | F |  |  |  | OI | F | F | T | T | GH, Gn, TSH | NA | NA | NA | V |  | T | 2540 | F | 37.3 | F | T | NA | NA |
| 8 | Aulinas 2021 | 8 | 24 | 27 | F | 30 | AGENESIS | F | F |  |  |  | OI | F | F | T | T | GH, Gn, TSH | NA | NA | NA | CS | T |  | 3260 | F | 40.6 | F | T | NA | NA |
| 8 | Aulinas 2021 | 10 | 25 | 28 | F | 36 | CRANIOPHARYNGIOMA | T | F |  |  |  | IVF | T | T | T | T | GH, Gn, TSH, ACTH, AVP-D | NA | NA | NA | CS | F |  | 3480 | M | 40 | F | T | NA | NA |
| 8 | Aulinas 2021 | 11 | 26 | 29 | F | 35 | ADENOMA | T | F |  |  |  | IVF | T | T | T | T | GH, Gn, TSH, AVP-D, ACTH | NA | NA | NA | V |  | T | 3430 | M | 40 | F | F | NA | NA |
| 8 | Aulinas 2021 | 12 | 27 | 30 | F | 42 | CUSHING | T | T |  |  |  | S | F | T | T | T | GH, TSH, ACTH partial | NA | NA | NA | V |  | T | 2790 | M | 40.5 | F | F | NA | NA |
| 9 | Sowithayasakul 2021 | 1 | 28 | 31 | F | 22 | CRANIOPHARYNGIOMA | T | F | 1 | 1 | 0 | S | F | F | F | F | None | NA | NA | NA | V |  | F | 2880 | M | 38 | NA | T | NA | NA |
| 9 | Sowithayasakul 2021 | 2a | 29 | 32 | F | 39 | CRANIOPHARYNGIOMA | T | F | 1 | 1 | 0 | OI | T | T | T | F | H, Thy, De | NA | NA | NA | V |  | F | 2660 | F | 38 | NA | T | NA | NA |
| 9 | Sowithayasakul 2021 | 2b | 29 | 33 | F | 41 | CRANIOPHARYNGIOMA | T | F | 2 | 2 | 0 | OI | T | T | T | F | H, Thy, De | NA | NA | NA | V |  | F | 2960 | F | 38.6 | NA | T | NA | NA |
| 9 | Sowithayasakul 2021 | 3 | 30 | 34 | F | 38 | CRANIOPHARYNGIOMA | T | F | 1 | 1 | 0 | OI | T | T | T | F | Insulin, H, Thy, De | NA | NA | NA | CS |  |  | 3520 | F | 37.2 | NA | F | NA | NA |
| 9 | Sowithayasakul 2021 | 4a | 31 | 35 | F | 30 | CRANIOPHARYNGIOMA | T | F | 1 | 1 | 0 | S | F | F | F | F | None | NA | NA | NA | CS |  |  | 3000 | M | 40 | NA | T | NA | NA |
| 9 | Sowithayasakul 2021 | 4b | 31 | 36 | F | 36 | CRANIOPHARYNGIOMA | T | F | 2 | 2 | 0 | S | F | F | F | F | None | NA | NA | NA | CS |  |  | 3000 | F | 41 | NA | T | NA | NA |
| 9 | Sowithayasakul 2021 | 5a | 32 | 37 | T | 28 | CRANIOPHARYNGIOMA | T | F | 1 | 1 | 0 | OI | T | T | T | F | H, Thy, De | NA | NA | NA | CS |  |  | 2270 | F | 34 | T | F | NA | NA |
| 9 | Sowithayasakul 2021 | 5b | 32 | 38 | T | 28 | CRANIOPHARYNGIOMA | T | F | 1 | 1 | 0 | OI | T | T | T | F | H, Thy, De | NA | NA | NA | CS |  |  | 2350 | M | 34 | NA | F | NA | NA |
| 9 | Sowithayasakul 2021 | 6a | 33 | 39 | F | 27 | CRANIOPHARYNGIOMA | F | F | 1 | 1 | 0 | S | F | F | F | F | None | NA | NA | NA | V |  | F | 3800 | M | 43 | NA | F | NA | NA |
| 9 | Sowithayasakul 2021 | 6b | 33 | 40 | F | 29 | CRANIOPHARYNGIOMA | T | F | 2 | 2 | 0 | S | NA | NA | NA | NA | ** | NA | NA | NA | CS |  |  | 2800 | F | 36 | NA | F | NA | NA |
| 10 | Kübler 2009 | 1 | 34 | 41 | F | 35 | CRANIOPHARYNGIOMA | T | T | 1 | 1 | 0 | IVF | T | T | T | F | H, Thy, De, OT | NA | NA | NA | CS | F |  | 2950 | F | 38.6 | T | F | T | NA |
| 11 | Correa 2017 | 1 | 35 | 42 | F | 25 | CHPD | F | F |  |  |  | OI | F | F | T | T | GH, Thy | NA | NA | NA | CS |  |  | 3365 | NA | 39.4 | F | T | NA | NA |
| 11 | Correa 2017 | 2 | 36 | 43 | F | 27 | CHPD | F | F |  |  |  | S | T | T | T | F | Pred, De, Thy | NA | NA | NA | CS |  |  | 3270 | NA | 38.5 | T | F | NA | NA |
| 11 | Correa 2017 | 3 | 37 | 44 | F | 26 | CHPD | F | F |  |  |  | IVF | F | T | T | T | GH,Pred, Thy | NA | NA | NA | V |  |  | 2870 | NA | 38 | F | T | NA | NA |
| 11 | Correa 2017 | 4 | 38 | 45 | F | 35 | CHPD | F | F |  |  |  | IVF | T | F | T | F | De, Thy | NA | NA | NA | CS |  |  | 1650 | NA | 35 | T | T | NA | NA |
| 11 | Correa 2017 | 5a | 39 | 46 | T | 37 | CHPD | F | F |  |  |  | IVF | F | T | T | T | GH, Pred, Thy | NA | NA | NA | V |  |  | 2740 | NA | 36 | F | F | NA | NA |
| 11 | Correa 2017 | 5b | 39 | 47 | T | 37 | CHPD | F | F |  |  |  | IVF | F | T | T | T | GH, Pred, Thy | NA | NA | NA | V |  |  | 2400 | NA | 36 | F | F |  |  |
| 12 | Gemzell 1973 | 2a | 40 | 48 | T | 26 | ACROMEGALY | F | F |  |  |  | OI | F | F | F | F | None | NA | NA | NA | V |  | F | 3400 | NA | TE | F | T | NA | NA |
| 12 | Gemzell 1973 | 2b | 40 | 49 | T | 26 | ACROMEGALY | F | F |  |  |  | OI | F | F | F | F | None | NA | NA | NA | V |  | F | 3200 | NA | TE | F | T |  |  |
| 12 | Gemzell 1973 | 3a | 41 | 50 | T | 28 | CHROMOPHOBE ADENOMA | T | T |  |  |  | OI | F | F | T | F | Thy | NA | NA | NA | CS | T | T | 2800 | NA | 40 | F | F | NA | NA |
| 12 | Gemzell 1973 | 3b | 41 | 51 | T | 28 | CHROMOPHOBE ADENOMA | T | T |  |  |  | OI | F | F | T | F | Thy | NA | NA | NA | CS | T | T | 2500 | NA | 40 | F | F |  |  |
| 12 | Gemzell 1973 | 4 | 42 | 52 | F | 34 | CHROMOPHOBE ADENOMA | T | T |  |  | 2 | OI | T | T | T | F | Thy, Cort | NA | NA | NA | CS | F |  | 3500 | M | TE | F | F | NA | NA |
| 12 | Gemzell 1973 | 5a | 43 | 53 | T | 29 | CHROMOPHOBE ADENOMA | T | T |  |  |  | OI | F | T | T | F | Thy, Cort | NA | NA | NA | CS | F |  | 2700 | NA | 34 | F | F | NA | NA |
| 12 | Gemzell 1973 | 5b | 43 | 54 | T | 29 | CHROMOPHOBE ADENOMA | T | T |  |  |  | OI | F | T | T | F | Thy, Cort | NA | NA | NA | CS | F |  | 2800 | NA | 34 | F | F |  |  |
| 13 | J. Tzafettas 2005 | 1 | 44 | 55 | F | 33 | MACROADENOMA | T | F |  |  |  | OI | T | T | T | F | Cort, De, Thy | NA | ABNORMAL | ABNORMAL | CS | F |  | 3200 | F | 39 | F | F | NA | NA |
| 14 | M. Hayashi 2002 | 1 | 45 | 56 | F | 30 | CRANIOPHARYNGIOMA | NA | NA | 1 | 1 |  | OI | F | T | T | F | Corticosteroids, Thy | 4.5 | 15.1 | 1.3 | CS | T |  | 2764 | F | 40 | T | F | NA | NA |
| 15 | B. L. Cohen, 1980 | 1 | 46 | 57 | F | 29 | SHEEHAN | F | F | 3 | 2 | 1 | OI | F | T | T | F | H, Thy | <1 | NA | NA | CS | T |  | 2200 | F | 35 | F | F | NA | NA |
| 16 | Zhan 2021 | 1 | 47 | 58 | F | 29 | SHEEHAN | F | F | 2 | 1 | 1 | IVF | T | T | T | F | H, Thy, refused De | 1.52 | NA | NA | CS | F |  | 3550 | F | 37.6 | T | F | NA | NA |
| 17 | Polishuk 1965 | 1 | 48 | 59 | F | 27 | SHEEHAN | F | F | 2 | 2 | 0 | OI | F | T | T | F | Dexamethasone, Thy | NA | NA | NA | CS |  |  | 2170 | M | TE | F | F | NA | NA |
| 18 | Jørgensen 1973 | 1 | 49 | 60 | F | 27 | CHROMOPHOBE ADENOMA | T | F | 1 | 1 | 0 | OI | F | T | T | F | Prednisolone/dexamethasone, Thy | NA | NA | NA | V |  |  | 4010 | M | 38 | T | F | NA | NA |
| 19 | Verdú 1998 | 1 | 50 | 61 | F | 31 | LYMPHOCYTIC HYPOPHYSITIS | F | F |  |  |  | OI | T | T | T | F | H, Thy, De | 57 | NA | NA | CS | T |  | 2490 | F | 38 | F | T | NA | NA |
| 20 | Kodama 1986 | 1a | 51 | 62 | F | 25 | CRANIOPHARYNGIOMA | T | T | 1 | 1 | 0 | S | F | F | F | F | None | NA | NA | NA | V |  |  | 3100 | M | NA | F | F | NA | NA |
| 20 | Kodama 1986 | 1b | 51 | 63 | F | 28 | CRANIOPHARYNGIOMA | T | T | 2 | 2 | 0 | OI | F | F | F | F | None | 12.7 | NA | NA | V |  |  | 2340 | F | NA | F | F | NA | NA |
| 21 | Jain D 2013 | 1 | 52 | 64 | F | 25 | SHEEHAN | F | F | 2 | 1 | 1 | OI | F | F | F | F | None | 1.5 | NA | NA | V |  | T | 2750 | M | 37 | T | F | T | NA |
| 22 | Kaur 2019 | 1a | 53 | 65 | T | 27 | SHEEHAN | F | F |  |  |  | OI | F | T | T | F | Thy, prednisolone, Insulin | 2.6 | NA | NA | CS | F |  | 2100 | F | 36 | F | T | NA | NA |
| 22 | Kaur 2019 | 1b | 53 | 66 | T | 27 | SHEEHAN | F | F |  |  |  | OI | F | T | T | F | Thy, prednisolone, Insulin | 2.6 | NA | NA | CS | F |  | 2000 | F | 36 | F | T | NA | NA |
| 23 | Laway 2013 | 1a | 54 | 67 | F | 34 | SHEEHAN | F | F | 2 | 2 | 0 | S | F | T | T | F | Thy, prednisolone | 0.1263 | NA | NA | V |  |  | NA | NA | NA | T | T | None | None |
| 23 | Laway 2013 | 1b | 54 | 68 | F | 38 | SHEEHAN | F | F | 3 | 3 | 0 | S | NA | NA | NA | F | NA | 0.0613 | NA | NA | CS |  |  | NA | NA | NA | NA | T | None | None |
| 24 | Bhagat 2011 | 1 | 55 | 69 | F | 37 | SHEEHAN | F | F | 2 | 2 | 0 | S | F | T | T | F | Prednisolone, Thy | 10 | NA | NA | CS | F |  | 3000 | M | 38 | F | F | NA | NA |
| 25 | See 2005 | 1 | 56 | 70 | F | 38 | SHEEHAN | F | F | 3 | 2 | 1 | S | F | T | T | F | Prednisolone, Thy | 3.1 |  |  | V |  |  | NA | M | NA | F | F | NA | NA |
| 26 | Algu¨n 2003 | 1 | 57 | 71 | F | 38 | SHEEHAN | F | F | 2 | 2 |  | S | F | T | T | F | Prednisolone, Thy | 4.76 | 8.6 |  | V |  |  | NA | M | NA | NA | F | NA | NA |
| 27 | Kriplani 2000 | 1a | 58 | 72 | T | 31 | SHEEHAN | F | F | 2 | 2 |  | OI | F | T | T | F | Prednisolone, Thy | 4.5 | NA | NA | CS | T |  | 2000 | F | 34 | F | F | NA | NA |
| 27 | Kriplani 2000 | 1b | 58 | 73 | T | 31 | SHEEHAN | F | F | 2 | 2 |  | OI | F | T | T | F | Prednisolone, Thy | 4.5 | NA | NA | CS | T |  | 1900 | M | 34 | F | F | NA | NA |
| 28 | Giustina 1985 | 1 | 59 | 74 | F | 25 | SHEEHAN | F | F | 3 | 3 |  | S | F | T | F | F | Cortisone acetate | 0.022 | NA | NA | V |  | F | NA | M | TE | NA | F | NA | NA |
| 29 | Moreira 1984 | 1a | 60 | 75 | F | 20 | SHEEHAN | F | F | 2 | 2 | 0 | S | F | F | F | F | None | NA | NA | NA | V |  |  | 3950 | M | TE | F | F | NA | NA |
| 29 | Moreira 1984 | 1b | 60 | 76 | F | 29 | SHEEHAN | F | F | 5 | 3 | 2 | S | F | T | T | F | Pred, Thy | NA | NA | NA | V |  | F | 3800 | NA | TE | F | F | NA | NA |
| 29 | Moreira 1984 | 1c | 60 | 77 | F | 30 | SHEEHAN | F | F | 6 | 4 | 2 | S | F | T | T | F | Pred, Thy | 14.5 | NA | NA | V |  | F | NA | F | TE | F | F | NA | NA |
| 30 | Jialal 1984 | 1 | 61 | 78 | F | 35 | SHEEHAN | F | F | 3 |  |  | OI | F | T | T | F | Cortisone acetate, Thy | 2.3 | 25 |  | CS |  |  | NA | M | 38 | NA | F | NA | NA |
| 31 | Grimes 1980 | 1 | 62 | 79 | F | 38 | SHEEHAN | F | F | 10 | 3 | 7 | S | F | T | T | F | Cortisone acetate, Thy | 15 |  |  | V |  | F | 1588 | M | 32 | F | F | NA | NA |
| 32 | Bowers 1974 | 1 | 63 | 80 | F | 21 | SHEEHAN | F | F | 2 | 2 |  | S | F | T | T | F | Pred, Thy | NA | NA | NA | V |  |  | NA | F | TE | F | F | NA | NA |
| 33 | Martin 1970 | 1 | 64 | 81 | F | 35 | SHEEHAN | F | F | 6 | 5 | 1 | S | F | T | T | F | Cort, Thy | NA | NA | NA | CS |  |  | 2523 | F | NA | T | F | NA | NA |
| 34 | Jackson 1969 | 1 | 65 | 82 | F | 37 | SHEEHAN | F | F | 2 | 2 |  | S | F | F | F | F | None | NA | NA | NA | V |  |  | NA | M |  | NA | F | NA | NA |
| 34 | Jackson 1969 | 2 | 66 | 83 | F | 34 | SHEEHAN | F | F | 3 | 3 |  | S | F | T | F | F | Cortisone | NA | NA | NA | V |  | T | NA | NA | NA | F | F | Stilbestrol* | None |
| 35 | Voutetakis 2004 | A | 67 | 84 | F | 24 | GENETIC | F | F | 1 | 1 | 0 | OI | F | F | T | F | Thy | NA | 21 | NA | CS |  |  | 3000 | NA | NA | F | F | NA | NA |
| 35 | Voutetakis 2004 | B | 68 | 85 | F | 28 | GENETIC | F | F | 1 | 1 | 0 | OI | F | F | T | F | Thy | NA | 8 | NA | V |  |  | 3700 | NA | NA | F | F | NA | NA |
| 35 | Voutetakis 2004 | B | 68 | 86 | F | 32 | GENETIC | F | F | 2 | 2 | 0 | OI | F | F | T | F | Thy | NA | NA | NA | V |  |  | 3000 | F | TE | F | F | NA | NA |
| 36 | De Coopman 1992 | 1 | 69 | 87 | F | 20 | ABSCESS | T | F | 2 | 2 |  | S | T | T | T | F | De, Thy, Cort | NA | NA | NA | V |  | F | 3997 | F | TE | F | T | NA | NA |

Abbreviations: AVP-D - arginine vasopressin deficiency, Br - bromocriptine, Cort - cortisol, CS - cesarean section, De - desmopressin, F - false (or female, in SEX column), GH - Growth hormone, GL - glucocorticoids, Gn - gonadotropin, GPA - gravida/para/abortus, H - hydrocortisone, IVF - in vitro fertilization, IUI - intrauterine insemination, M - male, NA - not available, OI - ovulation induction, OT - Oxytocin, PCOS – polycystic ovary syndrome, Pred - prednisone, S - spontaneous, T - true, TE - term, Thy - Thyroxine, V - vaginal

*Stilbestrol was started to suppress any possible lactation in view of the previous history of tuberculosis.

**Subsequently developed panhypopituitarism and required hormonal supplementation
